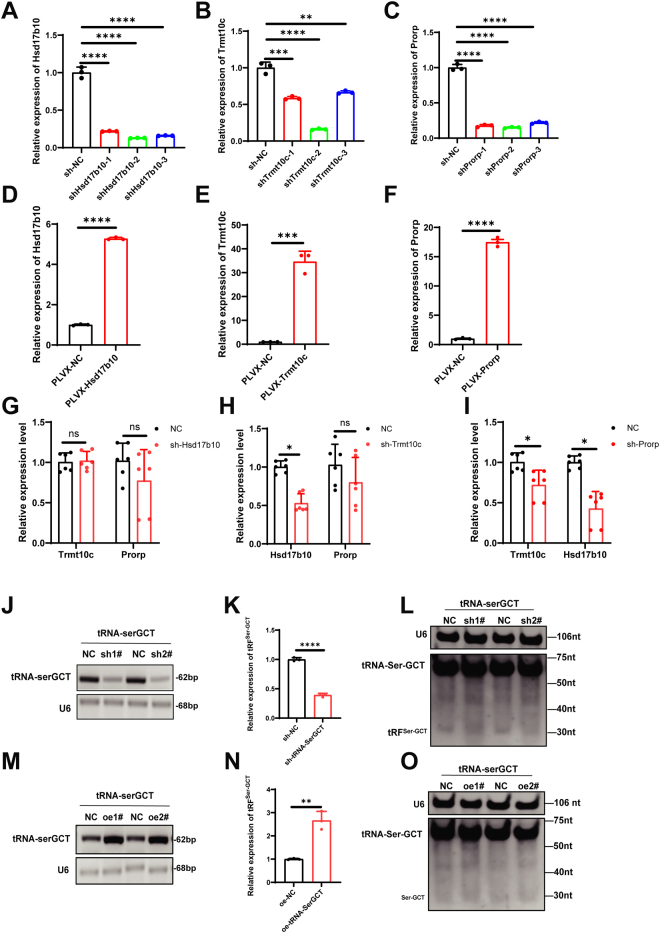# RNase P generated tRF^Ser-GCT^ promotes fat storage in adipocytes *via* Adrb2 signaling

**DOI:** 10.1016/j.jbc.2025.110820

**Published:** 2025-10-14

**Authors:** Linyuan Shen, Yuhang Lei, Xue Zhao, Xinyi Wang, Dujun Chen, Kai Wang, Yi Zhong, Tianci Liao, Yiting Yang, Lei Chen, Ye Zhao, Lili Niu, Xiaofeng Zhou, Mailin Gan, Li Zhu

**Affiliations:** 1 Farm Animal Germplasm Resources and Biotech Breeding Key Laboratory of Sichuan Province, Sichuan Agricultural University, Chengdu, China; 2 State Key Laboratory of Swine and Poultry Breeding Industry, Sichuan Agricultural University, Chengdu, China; 3 Key Laboratory of Livestock and Poultry Multi-omics, Ministry of Agriculture and Rural Affairs, College of Animal and Technology, Sichuan Agricultural University, Chengdu, China; 4 Key Laboratory of Bio-Resource and Eco-Environment of Ministry of Education, Animal Disease Prevention and Food Safety Key Laboratory of Sichuan Province, College of Life Sciences, Sichuan University, Chengdu, China

**Keywords:** Adrb2, Hsd17b10, lipid accumulation, RNase P, tRF^Ser-GCT^

## Abstract

tRNA-derived small RNAs (tsRNAs) are emerging regulators of metabolism, but their roles in adipose tissue are not well defined. Here, we profiled tsRNA expression in mouse brown adipose tissue (BAT), gonadal white adipose tissues, and inguinal white adipose tissue (iWAT), revealing depot-specific patterns and a notable enrichment of mitochondrial tRF-5c fragments, especially tRF-1:29-chrM.Ser-GCT (tRF^Ser-GCT^), in BAT. tRF^Ser-GCT^ expression correlated with mitochondrial abundance and increased during adipogenic differentiation and metabolic activation, both *in vivo* and *in vitro*. Functionally, tRF^Ser-GCT^ promoted adipogenesis and lipid accumulation in 3T3-L1 cells and localized to mitochondria. Mechanistically, tRF^Ser-GCT^ is generated from mitochondrial tRNA^Ser-GCT^ by the Ribonuclease P complex (Trmt10c, Hsd17b10, Prorp), with Hsd17b10 being essential for its biogenesis and for normal adipocyte lipid metabolism. tRF^Ser-GCT^ directly targets and downregulates Adrenoceptor Beta 2, a key metabolic regulator. *In vivo*, restoring tRF^Ser-GCT^ in Hsd17b10-deficient mice partially rescued adipose tissue accumulation and adipogenic gene expression. Together, these findings identify tRF^Ser-GCT^ as a mitochondrial tsRNA that promotes adipogenesis *via* the Ribonuclease P – Adrenoceptor Beta 2 axis, revealing a new layer of tsRNA-mediated regulation in adipose tissue biology.

Fat is an important part of the body and it plays a role that cannot be ignored in the body's metabolism (1). Adipogenesis is the major pathway for the formation of adipose tissue and involves the proliferation and differentiation of preadipocytes (2). White adipose tissue (WAT), as the main part of the body's energy storage, is responsible for storing excess energy in the form of triglycerides (TGs) (3, 4). Excessive deposition of WAT can lead to obesity and increase the risk of metabolic disorders, including cardiovascular disease, type 2 diabetes and nonalcoholic fatty liver disease (5). The *in vitro* studies of preadipocyte development help us to explore the molecular mechanisms of adipogenesis and lipid accumulation, and to a certain extent contribute to the prevention and treatment of obesity and other metabolic diseases. Accumulating evidence indicates that tRNA-derived fragments (tsRNAs) are widely expressed, functionally versatile, and play important roles in the regulation of diverse biological processes. For example, sperm tsRNA may mediate the intergenerational inheritance of diet-induced metabolic disorders (6), 5′ tsRNA leads to translation shutdown by replacing the m7G cap-binding protein eIF4E of mRNAs, tsRNA-06018 promotes adipogenesis (7, 8). These findings highlight the importance of elucidating the regulatory mechanisms of tsRNAs in adipogenesis.

tRFs are small noncoding RNAs derived from tRNA and cleaved by transfer RNA (tRNA) in different ways (8, 9, 10). tRFs were first discovered in prokaryotes, and later studies have found that they are widespread in eukaryotic mammals and may produce more tRFs in the event of stress reactions (hypoxia, oxidation, viral infection) (11). According to the position of tRFs on primary or mature tRNA transcripts, they can be divided into: tRF-5s, tRF-3s and tRF-1s tRF-5s and tRF-3s are generated from the 5′ and 3′ ends of mature tRNAs, whereas tRF-1s are generated from the 3′ ends of primary tRNA transcripts (10, 12). tRF-1:29-chrM.Ser-GCT (tRF^Ser-GCT)^ is derived from mitochondrial tRNA12-SerGCT, 29 bases long, and belongs to tRF-5c in tRF-5s. The full length of tRF-5s is 14 to 30 bases. It is cut from the D loop of the tRNA gene or the region between the D loop and the anticodon loop. According to the specific length, it is further divided into tRF-5a (14–16 nt), tRF-5b (22–24 nt) and tRF-5c (28–30 nt), which have been reported in mammalian cells, plants and fission yeast (12, 13, 14, 15). Recent studies have shown that this novel noncoding RNA is involved in epigenetic regulation at the pre-transcriptional and post-transcriptional levels of the body, regulating the expression of target genes through sequence complementation (16, 17). In addition, tRFs also have biological functions such as inhibiting the translation process and regulating the cell cycle (18, 19). Although there are few reports on tRFs, a novel epigenetic factor in the development and formation of adipose tissue, as an important new member of the epigenetic regulatory network, its functions in adipogenesis and lipid accumulation and role cannot be ignored. Our previous sequencing of mice identified some new potential tRFs, including tRF^Ser-GCT^, which is highly expressed in adipose tissue. However, whether tRF^Ser-GCT^ plays a role in adipogenesis and lipid deposition, and how it functions, remains unclear (20).

Mitochondrial tRNAs (mtRNAs) differs from cytoplasmic tRNAs, in general, mtRNAs are shorter than cytoplasmic tRNAs, mtRNAs are 59 to 75 nucleotides in length, while cytoplasmic tRNAs are 76 to 93 nucleotides in length (21). In addition, some mtRNAs, such as mtRNA-Ser and mtRNA-Lys, have smaller stems and loops, and some are even missing. Ribonuclease P (RNase P), as a ribonucleoprotein or pure protein form, catalyzes the removal of the 5′ leader from the precursor tRNA (22, 23, 24). Mitochondrial RNase P is a protein complex that does not contain catalytic RNA and consists of three proteins: mitochondrial ribonuclease P protein 1 (MRPP1, also known as tRNA methyltransferase 10c homolog, Trmt10c), MRPP2 (also known as hydroxysteroid) 17-β dehydrogenase 10, Hsd17b10), and MRPP3 (also known as the protein only RNase P catalytic subunit, Prorp), all three proteins are required for RNase P activity (25, 26, 27). In tRNA cleavage, not only the cleavage at the loop position, but also the occurrence of cleavage independent of the loop position, such as cleavage by RNase P targeting a specific tRNA stem position (28). Since the tRNA derived from tRF^Ser-GCT^ belongs to the mtRNA-Ser category, its D arm is deleted, and the cleavage site of tRF^Ser-GCT^ happens to be located at the anticodon stem, so we speculate that tRF^Ser-GCT^ may be caused by RNase P Cleaved at the position of the targeted tRNA anticodon stem.

In this study, we investigated the role of tRF^Ser-GCT^ in adipogenesis and lipid accumulation, and further explored the specific regulatory mechanism of tRF^Ser-GCT^ in promoting adipogenesis and lipid deposition.

## Results

### tsRNA expression characterization in diverse mouse adipose compartments

**Figure 1:**
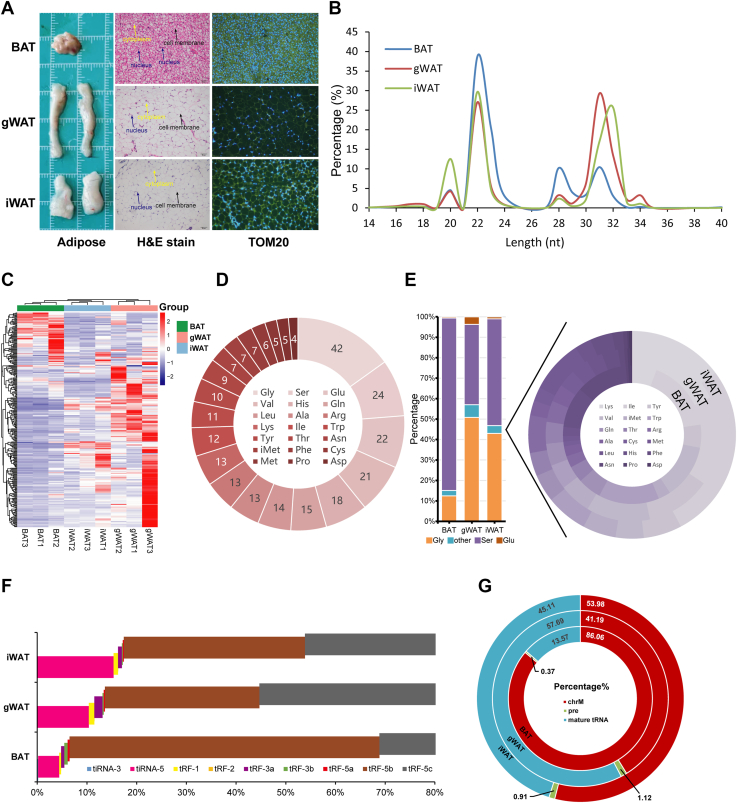
**Characterization of tsRNAs in mouse adipose tissues.***A*, morphological and histological analyses of BAT, gWATand iWAT in mice (n = 5). *Left*: gross tissue morphology; middle: H&E staining; *right*: immunofluorescence staining of TOM20, a mitochondrial marker. This scale bar represent for H&E staining and immunofluorescence staining. The scale bar represent 50 μm. *B*, length distribution of tRNA-derived small RNA (tsRNAs) in BAT, gWAT, and iWAT. *C*, heatmap clustering analysis showing differential expression patterns of tsRNAs among differential adipose. *D*, total count of tsRNAs derived from various parental tRNAs. *E*, proportional distribution of tsRNAs based on their parental tRNA types across different adipose tissues. *F*, classification of tsRNA subtypes in BAT, epididymal white adipose tissue , and iWAT. *G*, composition of tsRNA origins categorized by mature nuclear tRNAs (tRNA), precursor tRNAs (pre), and mitochondrial tRNAs (chrM). BAT, brown adipose tissue; gWAT, gonadal white adipose tissue; iWAT, inguinal white adipose tissue; tsRNA, tRNA-derived small RNA.

We examined the morphology and mitochondrial abundance of BAT, gWAT, and iWAT in mice. H&E staining showed multilocular adipocytes in BAT, while gWAT and iWAT displayed typical unilocular white adipocytes. Immunofluorescence staining of TOM20 revealed a high mitochondrial abundance in BAT, with markedly lower levels in white adipose depots (gWAT and iWAT). Among these white depots, iWAT displayed stronger TOM20 immunofluorescence than gWAT in our samples (Fig. 1*A*). Notably, mitochondrial abundance varied significantly among these adipose depots and was positively correlated with the tissue metabolic demand. We analyzed the length distribution of tsRNAs in BAT, gWAT, and iWAT. All three adipose tissues exhibited two main peaks, centered on 22 nucleotides and 31 nucleotides, corresponding to typical lengths of tRF-5 and tRF-3 fragments, respectively. Notably, BAT showed a prominent enrichment of tsRNAs at ∼22 nt, whereas gWAT and iWAT displayed relatively higher proportions of longer tsRNAs (∼31 nt) (Fig. 1*B*). Heatmap analysis revealed distinct tsRNA expression profiles among BAT, iWAT, and gWAT. Clustering patterns indicated tissue-specific expression signatures, with BAT and gWAT displaying the most divergent tsRNA profiles (Fig. 1*C*). Furthermore, we analyzed the distribution of tsRNAs derived from different parental tRNAs. Glycine (Gly)-tRNA-derived tsRNAs were the most abundant across all adipose tissues, followed by tsRNAs originating from Ser, Glu, and Val tRNAs, suggesting a preferential processing pattern from specific tRNA species (Fig. 1*D*). To further characterize the origin of tsRNAs in different adipose tissues, we analyzed the relative proportions of tsRNAs derived from individual tRNA species across BAT, gWAT, and iWAT. In BAT and iWAT, tsRNAs were predominantly derived from Ser-tRNAs, whereas Gly-tRNA-derived tsRNAs were enriched in gWAT. These findings suggest tissue-specific preferences in tsRNA biogenesis from distinct tRNA sources (Fig. 1*E*). We characterized the distribution of tsRNA subtypes in BAT, gWAT, and iWAT. tRF-5b was the most abundant subtype in BAT, while tRF-5c was the most abundant in both gWAT and iWAT. Other subtypes such as tRF-1, tRF-3, and tiRNAs were present at lower proportions, with subtle differences observed among the three fat depots, indicating tissue-specific preferences in subtype distribution (Fig. 1*F*). We further characterized the origin of tsRNAs based on their parental tRNA types, including mitochondrial tRNAs (chrM), pre-tRNAs, and mature tRNAs. The majority of tsRNAs in iWAT and gWAT originated from mature tRNAs, with proportions of 45.11% and 53.98%, respectively, whereas BAT showed a lower proportion of 13.57% mature tRNAs. Pre-tRNA- and mitochondrial tRNA-derived tsRNAs accounted for smaller fractions (Fig. 1*G*). In summary, these results suggest that tsRNAs exhibit distinct expression patterns across different adipose tissues, with variations in length distribution, subtype composition, and parental tRNA origin. These differences correlate with tissue-specific metabolic characteristics and suggest potential regulatory roles of tsRNAs in adipose tissue function and identity.

### Identification and mitochondrial characterization analysis of tRF^Ser-GCT^

**Figure 2:**
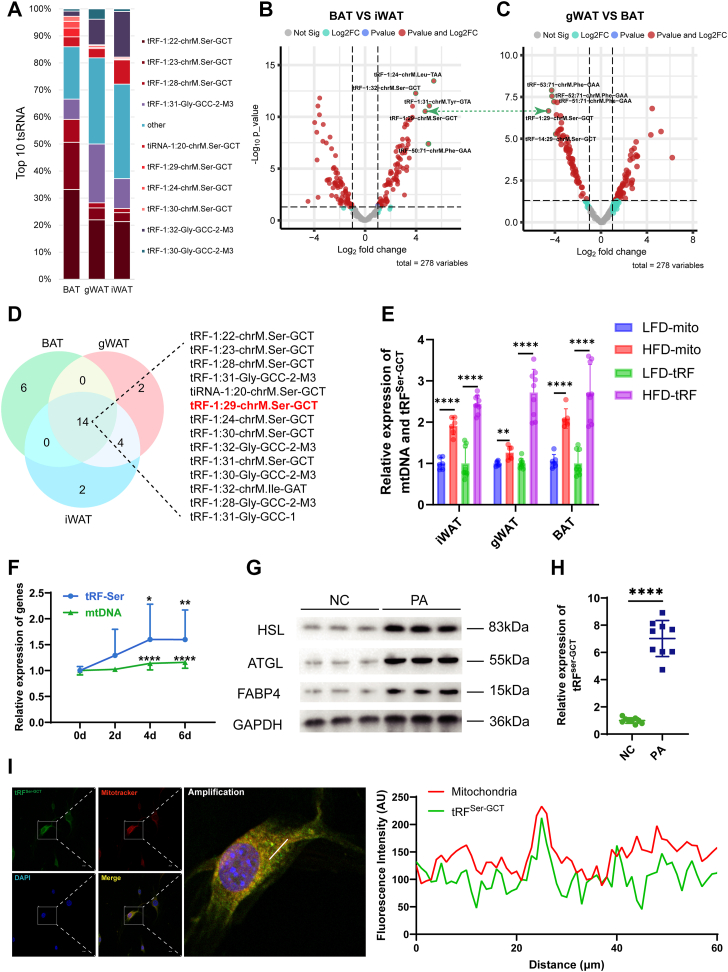
**Identification and mitochondrial characterization analysis of tRF^Ser-GCT^.***A*, stacked bar plots show the top 10 most highly expressed tsRNAs in BAT, gWAT, and iWAT. *B*, volcano plot of differentially expressed tsRNAs between BAT and iWAT. *C*, volcano plot of differentially expressed tsRNAs between gWAT and BAT. *D*, venn diagram showing the overlap among the top 20 differentially expressed tsRNAs in BAT, gWAT, and iWAT. *E*, relative expression of mitochondrial DNA (mtDNA) and tRF^Ser-GCT^ in three adipose tissues under low-fat diet or high-fat diet (HFD) mice (n = 6). *F*, temporal expression of tRF^Ser-GCT^ and mtDNA during 3T3-L1 adipogenic differentiation (0 days, 2 days, 4 days, 6 days) (n = 9). *G*, Western blot analysis of lipid metabolism-related proteins (HSL, ATGL, FABP4) in 3T3-L1 adipocytes stimulated with palmitic acid (PA). NC, normally differentiated cells (no PA); PA, differentiated cells treated with PA. *H*, expression level of tRF^Ser-GCT^ in 3T3-L1 cells following PA treatment. *I*, subcellular localization of tRF^Ser-GCT^ in 3T3-L1 cells as assessed by Fluorescence In Situ Hybridization and mitotracker staining. *Right panel* shows fluorescence intensity profiles quantifying partial overlap between tRF^Ser-GCT^ and mitochondria. The scale bar represent 20 μm. ∗*p* < 0.05, ∗∗*p* < 0.01, ∗∗∗*p* < 0.001, ∗∗∗∗*p* < 0.0001. All results above are representative of three independent experiments and presented as the means ± SD. BAT, brown adipose tissue; gWAT, gonadal white adipose tissue; iWAT, inguinal white adipose tissue; PA, palmitic acid; tsRNA, tRNA-derived small RNA.

To identify lipid metabolism-related tsRNAs, we performed differential expression analysis across BAT, gWAT, and iWAT based on tsRNA-seq data. The top 10 most abundant tsRNAs across BAT, gWAT, and iWAT were profiled. The composition and relative abundance of these tsRNAs varied notably among the three adipose depots. tRF^Ser-GCT^ was highly enriched in BAT, while the tsRNA composition exhibited notable differences between brown and white fat, indicating depot-specific expression patterns (Fig. 2*A*). We next performed differential expression analysis to identify specific tsRNAs among the three adipose tissues. Volcano plots revealed a number of significantly differentially expressed tsRNAs between BAT and iWAT (Fig. 2*B*), as well as between gWAT and BAT (Fig. 2*C*). In particular, the top five most significantly upregulated tsRNAs in BAT *versus* iWAT were highlighted, revealing several BAT-enriched candidates potentially associated with mitochondrial function, consistent with the high mitochondrial content of BAT. To identify shared tsRNA candidates across adipose tissues, we performed a Venn diagram comparison of the top 20 differentially expressed tsRNAs in BAT, gWAT, and iWAT (Fig. 2*D*). By integrating the results from differential expression analyses (Fig. 2, *B* and *C*) with the Venn diagram of the top 20 differentially expressed tsRNAs (Fig. 2*D*), we identified tRF^Ser-GCT^ as the only overlapping tsRNA across all comparisons. Given the significantly enriched expression of tRF^Ser-GCT^ in BAT and its mitochondrial origin, we hypothesized that its expression may correlate with mitochondrial abundance across adipose and dietary conditions. To further explore the relationship between tRF^Ser-GCT^ and mitochondrial content, we measured the expression levels of this tsRNA alongside mitochondrial DNA (mtDNA) in three adipose tissues under low-fat diet and high-fat diet (HFD) conditions. The result showed that the expression patterns of tRF^Ser-GCT^ closely mirrored the changes in mtDNA copy number across tissues and dietary groups (Fig. 2*E*).

To validate this association *in vitro*, we measured the expression levels of tRF^Ser-GCT^ and mitochondrial DNA during adipogenic differentiation of 3T3-L1 preadipocytes. During adipogenic differentiation of 3T3-L1 preadipocytes, the expression levels of tRF^Ser-GCT^ and mitochondrial DNA progressively increased, exhibiting highly similar temporal patterns (Fig. 2*F*). These *in vitro* findings were consistent with *in vivo* results from mouse adipose tissues, both demonstrating a positive correlation between tRF^Ser-GCT^ expression and mitochondrial abundance.

Correspondingly, to further examine whether tRF^Ser-GCT^ responds to metabolic activation, 3T3-L1 adipocytes were treated with palmitic acid (PA) to stimulate fatty acid metabolism. Western blot analysis confirmed successful induction of lipolytic activity, as indicated by elevated expression of HSL and ATGL, along with increased levels of FABP4 (Fig. 2*G*). Concurrently, the expression level of tRF^Ser-GCT^ was significantly upregulated upon PA treatment, suggesting that this tsRNA is responsive to fatty acid metabolic activation (Fig. 2*H*). To further examine the subcellular localization of tRF^Ser-GCT^, we performed fluorescence *in situ* hybridization in 3T3-L1 cells. Costaining with Mitotracker revealed partial colocalization of tRF^Ser-GCT^ with mitochondria, as shown in the merged images and the intensity profile analysis (right panel). These results provide multidimensional evidence supporting the mitochondrial origin of tRF^Ser-GCT^, confirming its localization within mitochondria at the subcellular level.

### RNase P regulates the expression of tRF^Ser-GCT^

**Figure 3:**
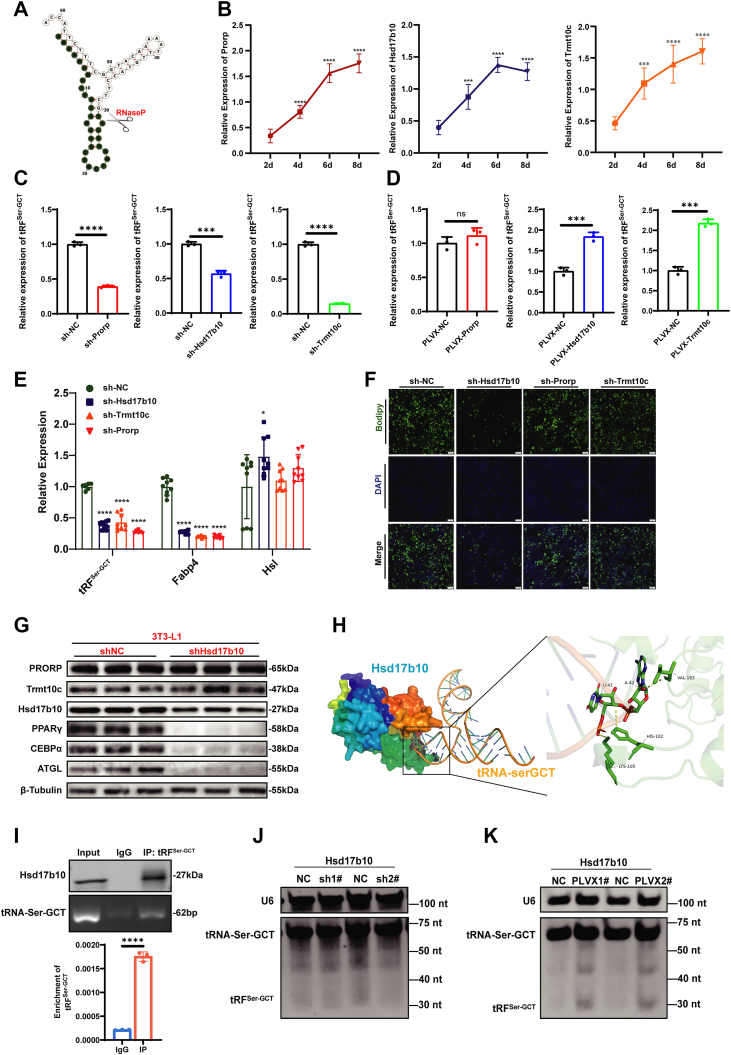
**Hsd17b10-dependent RNase P mediates the biogenesis of tRF^Ser-GCT^ and regulates adipocyte lipid metabolism.***A*, tRF^Ser-GCT^ shear diagram. *B*, qRT-PCR analysis of mRNA expression levels of Ribonuclease P subunits (Trmt10c, Hsd17b10, Prorp) during 3T3-L1 preadipocyte differentiation, normalized to GAPDH. *C*, qRT-PCR analysis of tRF^Ser-GCT^ levels after shRNA-mediated knockdown of Trmt10c, Hsd17b10, or Prorp in 3T3-L1 cells. sh-NC, non-targeting shRNA control; sh-Trmt10c/Hsd17b10/Prorp, gene-specific shRNAs. *D*, qRT-PCR analysis of tRF^Ser-GCT^ levels after overexpression of Trmt10c, Hsd17b10, or Prorp in 3T3-L1 cells. PLVX-NC, empty-vector control; PLVX-Prorp/PLVX-Hsd17b10/PLVX-Trmt10c, overexpression constructs. *E*, qRT-PCR analysis of tRF^Ser-GCT^, Fabp4, and Hsl expression in 3T3-L1 preadipocytes after knockdown of Hsd17b10, Prorp, or Trmt10c. *F*, BODIPY staining of lipid droplets in 3T3-L1 preadipocytes after knockdown of Hsd17b10, Prorp, or Trmt10c. *G*, Western blot analysis of PRORP, Trmt10c, Hsd17b10, PPARγ, C/EBPα, ATGL, and FABP4 protein levels in 3T3-L1 cells after Hsd17b10 knockdown. *H*, molecular docking model showing the binding interaction between tRNA-Ser-GCT and Hsd17b10. *I*, RIP-qPCR using an anti-Hsd17b10 antibody (IgG as negative control) showing enrichment of tRF^Ser-GCT^ in the Hsd17b10 IP relative to IgG. RT-PCR was performed to detect changes in the precursor tRNA^Ser-GCT^, while qRT-PCR was used to quantify the enrichment of tRF^Ser-GCT^ in the Hsd17b10 IP relative to IgG. Enrichment was normalized to input. *J*-*K*, northern blot analysis showing that Hsd17b10 knockdown decreases (*J*) and overexpression increases (*K*) tRF^Ser-GCT^ levels. ∗*p* < 0.05, ∗∗*p* < 0.01, ∗∗∗*p* < 0.001, ∗∗∗∗*p* < 0.0001. All results above are representative of three independent experiments and presented as the means ± SD.

tRF^Ser-GCT^ is derived from the 5′ end of mitochondrial tRNA chrM.tRNA12-SerGCT, with a length of 29 nucleotides, and is classified as a tRF-5c based on its origin and size (Fig. 3*A*). Given that tRF-5c fragments are typically generated by RNase P-mediated cleavage of precursor tRNAs (29, 30, 31), we next sought to determine whether the biogenesis of tRF^Ser-GCT^ in adipocytes depends on the activity of the mitochondrial RNase P complex. Mitochondrial RNase P is a protein complex that does not contain catalytic RNA and is composed of three proteins (Trmt10c, Hsd17b10, Prorp), all of which are required for RNase P activity. The dysfunction of any component of RNase P will affect RNA processing (31, 32). Firstly, we examined the expression trend of the mRNA levels of these three subunits during the differentiation of 3T3-L1 preadipocytes. The results showed that the mRNA expression levels of Trmt10c, Hsd17b10, and Prorp were up-regulated with the process of differentiation, which was consistent with the previous expression trend of tRF^Ser-GCT^ (Fig. 3*B*). To determine whether the cleavage activity of mitochondrial RNase P is required for tRF^Ser-GCT^ biogenesis, we designed the shRNA of Trmt10c, Hsd17b10, and Prorp. The knockdown efficiency of these shRNAs was determined by q-PCR (Fig. S1, *A*–*C*). We found that the depletion of Trmt10c, Hsd17b10, and Prorp all led to a significant reduction in tRF^Ser-GCT^ levels (Fig. 3*C*). Conversely, overexpression plasmids for Trmt10c, Hsd17b10, and Prorp were constructed to further validate their roles (Fig. S1, *D*–*F*). Overexpression of Hsd17b10 or Trmt10c significantly elevated tRFSer-GCT levels, whereas Prorp overexpression had no significant effect (Fig. 3*D*). Together with the loss-of-function data, these results indicate that mitochondrial RNase P contributes to tRFSer-GCT biogenesis, but gain-of-function effects are subunit-specific, suggesting that Prorp is not rate-limiting under our conditions or requires appropriate complex stoichiometry. In addition, we examined the interdependence among the three RNase P subunits by assessing the effects of knocking down each subunit on the expression of the other two. Knockdown of Hsd17b10 had no significant effect on Trmt10c or Prorp expression (Fig. S1*G*). In contrast, silencing Trmt10c led to a marked reduction in Hsd17b10 expression, but did not affect Prorp levels (Fig. S1*H*). Similarly, knockdown of Prorp significantly decreased the expression of both Trmt10c and Hsd17b10 (Fig. S1*I*).

To further clarify the functional significance of each RNase P subunit in tRF^Ser-GCT^ biogenesis and lipid metabolism, we examined the effects of knocking down Hsd17b10, Prorp, or Trmt10c in 3T3-L1 preadipocytes. Knockdown of Hsd17b10, Trmt10c, or Prorp in 3T3-L1 cells reduced tRF^Ser-GCT^ and Fabp4, with subunit-specific changes in Hsl; BODIPY imaging further showed decreased lipid droplet accumulation for all three knockdowns, with the largest reduction after Hsd17b10 depletion, indicating impaired adipogenic differentiation (Fig. 3, *E* and *F*). These findings highlight Hsd17b10 as a particularly critical subunit within the RNase P complex, exerting a dominant role in regulating tRFˆSer-GCT production and downstream adipogenic processes. At the protein level, knockdown of Hsd17b10 did not affect the expression of the other two RNase P subunits, PRORP and Trmt10c. However, depletion of Hsd17b10 led to a marked reduction in the levels of key lipid metabolism proteins, including PPARγ, C/EBPα, and ATGL (Fig. 3*G*). Molecular docking analysis revealed a clear binding interaction between tRNA^Ser-GCT^ and Hsd17b10 (Fig. 3*H*). This predicted interaction was further validated by RNA immunoprecipitation assays, which confirmed the physical association between tRF^Ser-GCT^ and Hsd17b10 in cells (Fig. 3*I*). northern blot analysis further confirmed the involvement of Hsd17b10 in the cleavage of tRNA^Ser-GCT^ to generate tRF^Ser-GCT^. Knockdown of Hsd17b10 resulted in a marked decrease in tRF^Ser-GCT^ levels, while overexpression of Hsd17b10 led to a significant increase in tRF^Ser-GCT^ abundance (Fig. 3, *J* and *K*). To further confirm the origin of tRF^Ser-GCT^, we manipulated the expression of its precursor tRNA^Ser-GCT^ in cells. Knockdown of tRNA^Ser-GCT^ was validated by RT-PCR (Fig. S1*J*), and resulted in a significant reduction in tRF^Ser-GCT^ levels as measured by qPCR (Fig. S1*K*) and northern blot (Fig. S1*L*). Conversely, overexpression of tRNA^Ser-GCT^ led to increased precursor levels (Fig. S1*M*), which was accompanied by a marked elevation of tRF^Ser-GCT^ as detected by both qPCR (Fig. S1*N*) and northern blot (Fig. S1*O*). These results provide direct evidence that tRF^Ser-GCT^ is generated from tRNA^Ser-GCT^ through a cleavage process.

Collectively, these results demonstrate that tRF^Ser-GCT^ is generated from tRNA^Ser-GCT^ through Hsd17b10-dependent RNase P-mediated cleavage, and that this process is essential for maintaining normal adipocyte lipid metabolism.

### Functional role of tRF^Ser-GCT^ in regulating adipogenic differentiation in 3T3-L1 cells

**Figure 4:**
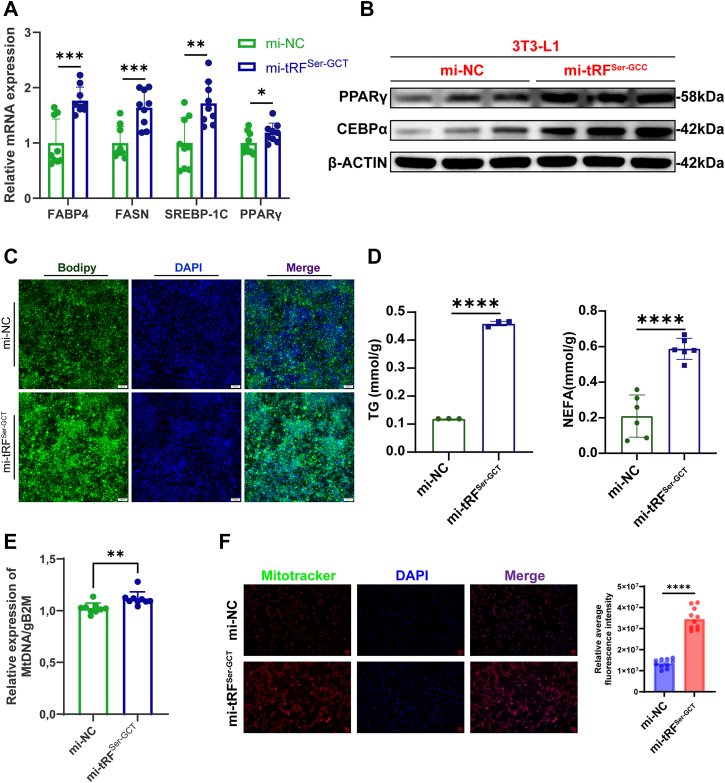
**Functional role of tRF^Ser-GCT^ in regulating adipogenic differentiation in 3T3-L1 cells.***A*, quantitative RT-PCR analysis of adipogenic and lipogenic gene expression in 3T3-L1 cells transfected with tRF^Ser-GCT^ mimic (mi-tRF^Ser-GCT^) or negative control (mi-NC). *B*, Western blot analysis of adipogenic proteins PPARγ and C/EBPα in 3T3-L1 cells after tRF^Ser-GCT^ overexpression. *C*, BODIPY staining of intracellular lipid droplets in 3T3-L1 cells. Scale: 100 μm. *D*, quantification of intracellular triglyceride and nonesterified fatty acid content in cells overexpressing tRF^Ser-GCT^. *E*, quantification of mtDNA copy number normalized to gB2M in 3T3-L1 cells. *F*, mitotracker staining showing mitochondrial content in 3T3-L1 cells. Quantification of fluorescence intensity is shown at *right*. This scale bar represent: 50 μm. ∗*p* < 0.05, ∗∗*p* < 0.01, ∗∗∗*p* < 0.001, ∗∗∗∗*p* < 0.0001. All results above are representative of three independent experiments and presented as the means ± SD. mtDNA, mitochondrial DNA

To investigate the potential role of tRF^Ser-GCT^ in adipocyte lipid metabolism, we overexpressed this tsRNA in 3T3-L1 preadipocytes and evaluated its effects through molecular, cellular, and biochemical analyses. Quantitative real-time PCR analysis revealed that overexpression of tRF^Ser-GCT^ significantly upregulated the expression of FABP4, FASN, SREBP-1C, and PPARγ compared to the negative control group (Fig. 4*A*). In line with the transcriptional upregulation of adipogenic markers, Western blot analysis showed increased protein expression of PPARγ and C/EBPα in cells overexpressing tRF^Ser-GCT^ compared to controls (Fig. 4*B*). Consistent with the upregulation of adipogenic markers, lipid accumulation was markedly increased in tRF^Ser-GCT^ overexpressing cells. Bodipy staining revealed enhanced intracellular lipid droplet formation compared to control cells (Fig. 4*C*), further supporting the role of tRF^Ser-GCT^ in promoting lipid storage. In addition, biochemical assays of intracellular TG and non-esterified fatty acid (NEFA) showed significant increases in cells overexpressing tRF^Ser-GCT^, indicating enhanced lipid synthesis/mobilization (Fig. 4*D*). Together, these data demonstrate that tRF^Ser-GCT^ functionally promotes adipogenesis and lipid metabolic activity in 3T3-L1 cells.

Given that tRF^Ser-GCT^ is of mitochondrial origin and that mitochondrial function is tightly linked to lipid metabolism, we further examined whether overexpression of this tsRNA influences mitochondrial abundance and activity in adipocytes. Quantitative PCR analysis revealed a significant increase in mtDNA copy number in tRF^Ser-GCT^ overexpressing cells, suggesting enhanced mitochondrial biogenesis (Fig. 4*E*). Consistently, mitotracker staining showed increased mitochondrial signal intensity in these cells, further confirming the elevation in mitochondrial content (Fig. 4*F*). In summary, these results suggest that tRF^Ser-GCT^ not only promotes adipogenic differentiation but may also enhance mitochondrial abundance in preadipocytes.

### tRF^Ser-GCT^ promotes adipogenic differentiation by targeting adrenoceptor beta 2 (Adrb2)

**Figure 5:**
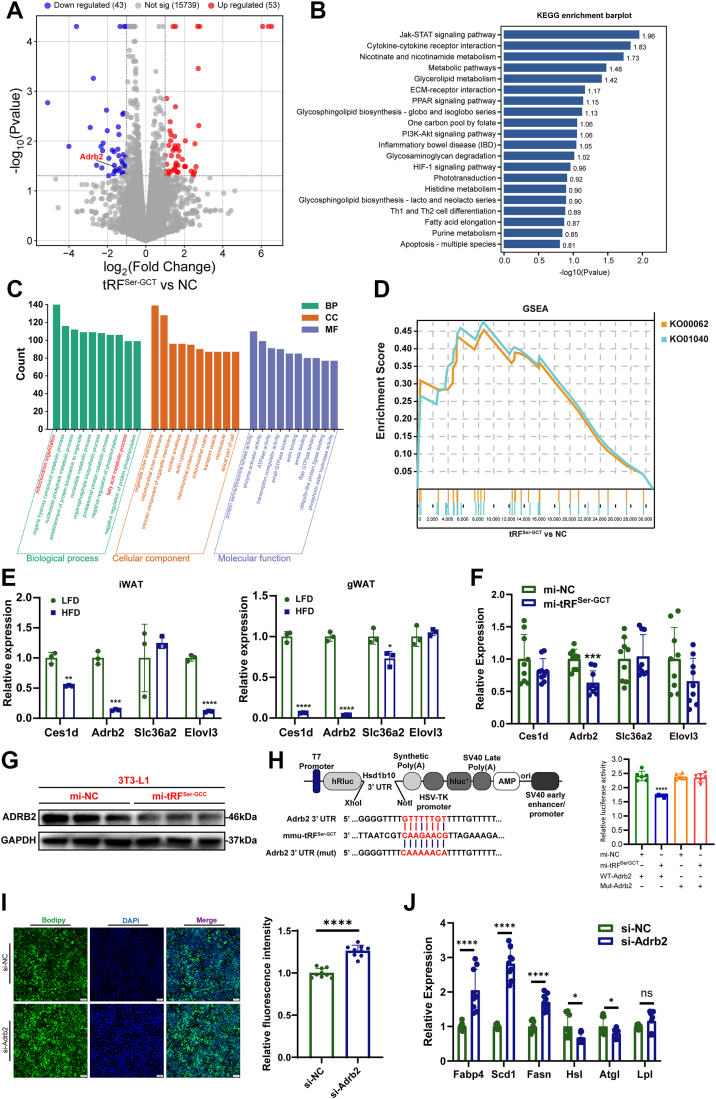
**tRF^Ser-GCT^ promotes adipogenic differentiation by targeting Adrb2.***A*, volcano plot of differentially expressed genes (DEGs) in 3T3-L1 cells transfected with tRF^Ser-GCT^ mimic *versus* negative control. *B*, Kyoto Encyclopedia of Genes and Genomes pathway enrichment analysis of DEGs identified in RNA-seq. *C*, gene ontology enrichment analysis of DEGs. *D*, gene set enrichment analysis showing significant enrichment of genes involved in fatty acid elongation (KO00062) and biosynthesis of unsaturated fatty acids (KO01040) in tRF^Ser-GCT^ overexpressing cells. *E*, qRT-PCR analysis of Ces1d, Adrb2, Slc36a2, and Elovl3 expression in gWAT and iWAT from mice fed HFD or low-fat diet . *F*, qRT-PCR analysis of candidate target genes in 3T3-L1 adipocytes overexpressing tRF^Ser-GCT^. *G*, Western blot analysis of Adrb2 protein levels in 3T3-L1 adipocytes transfected with tRF^Ser-GCT^ mimic or negative control. *H*, in *left*, schematic representation illustrating the predicted binding site of 5′ tRF^Ser-GCT^ with the seed region in the Adrb2 3′UTR, along with the corresponding mutation region. In *right*, dual-luciferase reporter assay validating the direct interaction between tRF^Ser-GCT^ and the 3′UTR of Adrb2. *I*, bodipy staining of lipid droplets in 3T3-L1 adipocytes following Adrb2 knockdown. The scale bar represent 100 μm. *J*, qRT-PCR analysis of adipogenic and lipogenic gene expression in 3T3-L1 adipocytes after Adrb2 knockdown. ∗*p* < 0.05, ∗∗*p* < 0.01, ∗∗∗*p* < 0.001, ∗∗∗∗*p* < 0.0001. All results above are representative of three independent experiments and presented as the means ± SD. DEG, differentially expressed gene.

To explore the molecular mechanism of tRF^Ser-GCT^ regulates adipogenic differentiation; we performed RNA-seq on 3T3-L1 cells transfected with tRF^Ser-GCT^ mimic or negative control. Differential expression analysis identified a total of 96 significantly altered genes, including 43 downregulated and 53 upregulated transcripts (Fig. 5*A*). Kyoto Encyclopedia of Genes and Genomes (KEGG) pathway analysis of differentially expressed genes (DEGs) revealed significant enrichment in several signaling and metabolic pathways, including metabolic pathways, glycerolipid metabolism, fatty acid corrosion, *etc.* (Fig. 5*B*). Complementary to the KEGG analysis, gene ontology (GO) enrichment was conducted to further dissect the functional impact of tRF^Ser-GCT^ associated transcriptomic changes. GO enrichment analysis revealed that DEGs were significantly enriched in terms such as mitochondrion organization, lipid acid metabolic process, and regulation of protein phosphorylation, underscoring the potential involvement of tRF^Ser-GCT^ in mitochondrial function and lipid metabolism (Fig. 5*C*). In addition, gene set enrichment analysis (GSEA) was performed to further assess the impact of tRF^Ser-GCT^ on metabolic pathways. The analysis revealed significant enrichment of genes involved in fatty acid elongation (KO00062) and biosynthesis of unsaturated fatty acids (KO01040) in tRF^Ser-GCT^ overexpressing cells (Fig. 5*D*).

To further investigate the mechanism by which tRF^Ser-GCT^ promotes lipid accumulation in adipocytes, we screened differentially expressed genes known to play critical roles in metabolic and lipid-related processes based on previous functional enrichment analyses. Using the binding site prediction tool RNAhybrid, we predicted potential interactions between tRF^Ser-GCT^ and these target genes. The analysis identified potential binding sites between tRF^Ser-GCT^ and several metabolic regulators, including Carboxylesterase 1 day (Ces1d), Adrenoceptor Beta 2 (Adrb2), Solute Carrier Family 36 Member 2 (Slc36a2), and ELOVL Fatty Acid Elongase 3 (Elovl3). To confirm the potential regulatory relationship between tRF^Ser-GCT^ and the predicted target genes, we examined the expression levels of Ces1d, Adrb2, Slc36a2, and Elovl3 in iWAT and gWAT from mice fed a HFD or normal chow (LFD). In iWAT, HFD significantly reduced Ces1d, Adrb2 and Elovl3, while Slc36a2 showed no significant change. In gWAT, HFD markedly reduced Ces1d and Adrb2, modestly decreased Slc36a2, and did not significantly alter Elovl3 (Fig. 5*E*). To further screen for potential target genes of tRF^Ser-GCT^, we examined the expression levels of Ces1d, Adrb2, Slc36a2, and Elovl3 in 3T3-L1 adipocytes overexpressing tRF^Ser-GCT^. Among these candidates, only Adrb2 showed a significant reduction in expression following tRF^Ser-GCT^ overexpression, while the other genes remained unchanged (Fig. 5*F*). Consistent with the mRNA findings, Western blot analysis demonstrated that overexpression of tRF^Ser-GCT^ in 3T3-L1 adipocytes led to a marked reduction in Adrb2 protein levels compared to the control group (Fig. 5*G*). Therefore, Adrb2 was selected as the primary candidate target for subsequent mechanistic investigations.

Furthermore, the direct interaction between tRF^Ser-GCT^ and Adrb2 was further validated using a dual-luciferase reporter assay. The 3′UTR of Adrb2 containing the predicted tRF^Ser-GCT^ binding site was cloned downstream of the luciferase gene. Cotransfection of tRF^Ser-GCT^ mimic with the wild-type Adrb2 3′UTR construct led to a significant reduction in luciferase activity, whereas mutation of the binding site abolished this effect (Fig. 5*H*). This result confirmed that tRF^Ser-GCT^ directly binds to the 3′UTR of Adrb2 and suppresses its expression at the post-transcriptional level. Further supporting the regulatory relationship between tRF^Ser-GCT^ and Adrb2, knockdown of Adrb2 in 3T3-L1 adipocytes produced effects similar to those observed with tRF^Ser-GCT^ overexpression. Bodipy staining revealed increased lipid droplet accumulation in cells transfected with siRNA targeting Adrb2 compared to negative controls (Fig. 5*I*). Consistently, the expression of key adipogenic and lipogenic genes, including Fabp4, Scd1, and Fas, was significantly upregulated following Adrb2 knockdown (Fig. 5*J*). Collectively, these findings demonstrate that tRF^Ser-GCT^ promotes adipogenic differentiation by directly targeting and downregulating Adrb2 in adipocytes.

### tRF^Ser-GCT^ regulates adipose tissue accumulation *in vivo via* the Hsd17b10–Adrb2 axis

**Figure 6:**
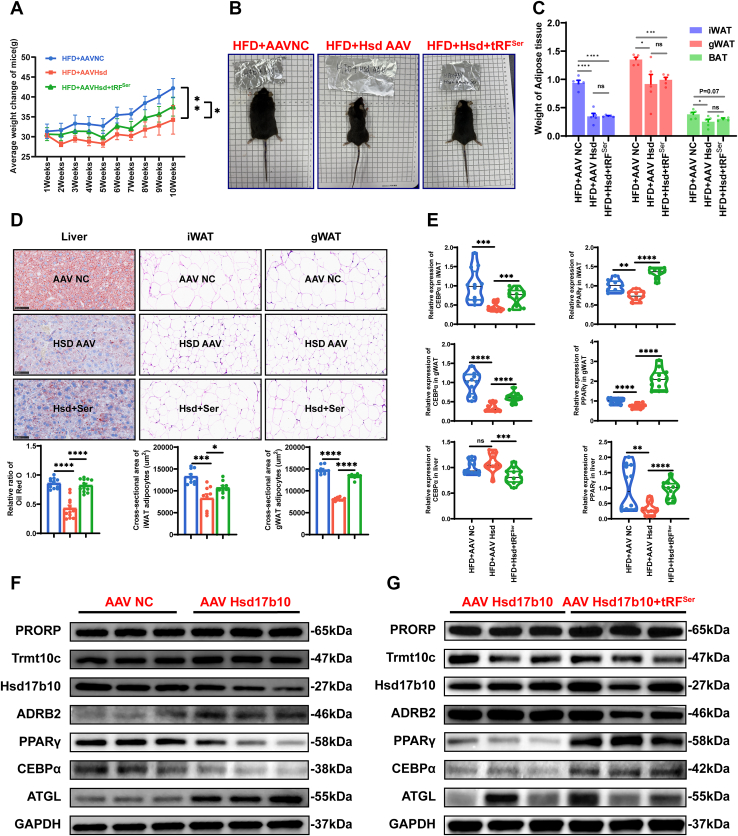
**tRF^Ser-GCT^ promotes adipogenic differentiation by targeting Adrb2.***A*, body weight changes in HFD mice treated with AAV-NC (control), AAV-Hsd (Hsd17b10 knockdown), or AAV-Hsd + tRF^Ser-GCT^ (tRF^Ser-GCT^ restoration group) over 10 weeks. Data are presented as mean ± SD, n = 5 mice per group. *B*, representative images of mice from each group showing differences in body size and adiposity after 10 weeks of treatment. *C*, the weight of iWAT, gWAT, and BAT in each group. *D*, histological analysis of liver, iWAT, and gWAT. Oil Red O staining of liver sections shows hepatic lipid accumulation; HE staining of iWAT and gWAT reveals adipocyte size. The scale bar represent 50 μm. *E*, qRT-PCR analysis of adipogenic transcription factors C/EBPα and PPARγ in liver, iWAT, and gWAT. *F*, Western blot analysis of PRORP, Trmt10c, Hsd17b10, Adrb2, PPARγ, C/EBPα, and ATGL protein levels in iWAT from AAV-NC and AAV-Hsd groups. *G*, Western blot analysis of the same proteins in iWAT from AAV-Hsd17b10 and AAV-Hsd + tRF^Ser-GCC^ groups. ∗*p* < 0.05, ∗∗*p* < 0.01, ∗∗∗*p* < 0.001, ∗∗∗∗*p* < 0.0001. All results above are representative of three independent experiments and presented as the means ± SD. gWAT, gonadal white adipose tissue; iWAT, inguinal white adipose tissue.

To investigate the *in vivo* role of tRF^Ser-GCT^ in adipose deposition, a HFD mouse model was established and subjected to different treatments: control (AAV-NC), Hsd17b10 knockdown (AAV-Hsd), and Hsd17b10 knockdown combined with tRF^Ser-GCT^ restoration (AAV-Hsd + tRF^Ser-GCT^). Hsd17b10 knockdown was achieved by AAV-mediated delivery, while tRF^Ser-GCT^ expression was restored using agomir injection. Mice with AAV-Hsd exhibited significantly reduced body weight gain compared to controls. However, restoration of tRF^Ser-GCT^ expression in Hsd17b10-deficient mice partially rescued the body weight phenotype, resulting in increased weight gain relative to AAV-Hsd group (Fig. 6*A*). Representative images of mice from each group further illustrate the differences in body size and adiposity (Fig. 6*B*). Consistent with the changes in body weight, the weights of iWAT, gWAT, and BAT were significantly reduced in the AAV-Hsd group compared to the AAV-NC group. However, restoration of tRF^Ser-GCT^ expression in Hsd17b10-deficient mice did not significantly rescue iWAT, gWAT, or BAT mass (Fig. 6*C*). Histological analysis was performed to further assess the effects of tRF^Ser-GCT^ on hepatic lipid accumulation and adipose tissue morphology. In the liver, Oil Red O staining showed reduced lipid accumulation in the AAV-Hsd group, which was partially restored upon tRF^Ser-GCT^ re-expression (Fig. 6*D*, left panel). HE staining of iWAT and gWAT revealed that Hsd17b10 knockdown resulted in a marked reduction in adipocyte size compared to controls, while restoration of tRF^Ser-GCT^ expression partially reversed this effect (Fig. 6*D*, middle and right panels). To further elucidate the molecular mechanisms underlying the observed phenotypic changes, the expression levels of key adipogenic transcription factors, C/EBPα and PPARγ, were quantified in liver, iWAT, and gWAT tissues. Both C/EBPα and PPARγ expression were significantly reduced in AAV-Hsd group mice compared to controls, while restoration of tRF^Ser-GCT^ expression partially rescued their expression levels in all examined tissues (Fig. 6*E*). Building on the transcriptional findings, Western blot analysis was performed to assess protein levels of lipid metabolism regulators, RNaseP subunits (PRORP, Trmt10c, Hsd17b10), and the tRF^Ser-GCT^ target gene Adrb2 in iWAT. In the AAV-Hsd group, expression of PRORP, Trmt10c, PPARγ, C/EBPα, and ATGL was reduced, while Adrb2 protein levels were elevated compared to controls (Fig. 6*F*). Restoration of tRF^Ser-GCT^ expression in Hsd17b10-deficient mice reversed these changes, resulting in increased levels of PRORP, Trmt10c, PPARγ, C/EBPα, and ATGL, and decreased Adrb2 expression (Fig. 6*G*). Collectively, these *in vivo* findings demonstrate that tRF^Ser-GCT^ promotes adipose tissue accumulation and adipogenic gene expression by modulating the Hsd17b10–Adrb2 regulatory axis.

## Discussion

A growing body of research shows that tRFs are involved in a variety of biological processes (9, 10). Under stress conditions, more tRFs may be produced, for example, the expression abundance of tRFs in chronic hepatitis and severe acute respiratory syndrome Coronavirus 2 (SARS-CoV-2) is significantly upregulated (33, 34). In our previous studies, it has been demonstrated that tRF^GluTTC^ can inhibit the differentiation of preadipocytes by reducing triglyceride content and lipid accumulation and reducing gene expression related to fatty acid synthesis (35). However, the role of tRF^Ser-GCT^ in preadipocytes has not been clearly defined. In this study, we found that tRF^Ser-GCT^ was upregulated during 3T3-L1 preadipocyte differentiation, consistent with the trend of PPARγ expression associated with lipogenesis. PA treatment, which simulates high-fat conditions, further increased tRF^Ser-GCT^ expression and the levels of lipogenesis-related genes, suggesting that tRF^Ser-GCT^ may play an important role in adipose development.

Mitochondrial RNase P, which is not a ribozyme, consists of three protein subunits and performs RNA cleavage and methylation by unknown mechanisms (36). The three protein subunits are MRPP1, MRPP2, and MRPP3 (encoded by Trmt10c, Hsd17b10, and Prorp, respectively) (25). In our study, knockdown of Trmt10c, Hsd17b10, and Prorp respectively reduced the expression of tRF^Ser-GCT^, possibly because the inactivation of RNase P reduced the shear of tRF^Ser-GCT^. However, only after knocking down Hsd17b10 did lipid deposition of adipocytes suffer the greatest impact, seriously hindering the transformation process of preadipocytes into adipocytes. Thus, we were able to determine that Hsd17b10 regulates preadipocyte differentiation at least in part by affecting tRF^Ser-GCT^ cleavage. Therefore, Hsd17b10 was chosen as the focus of the subsequent research.

The differentiation and maturation of preadipocytes are essential processes of lipid deposition. Functional experiments showed that tRF^Ser-GCT^ mimics promoted the differentiation of 3T3-L1 preadipocytes, as evidenced by increased lipid droplet formation and elevated expression of adipogenic markers at both mRNA and protein levels. Overexpression of tRF^Ser-GCT^ also led to higher TG and NEFA content, indicating enhanced lipid synthesis and storage. These results suggest that tRF^Ser-GCT^ is an important regulator of adipogenic differentiation. As far as the present study is concerned, tRFs function in a way similar to miRNA, which can regulate gene expression at the transcriptional level through interaction with the target gene. Normally, the expression level of miRNA is negatively correlated with the expression level of target genes. Mechanistically, our transcriptomic and functional analyses identified Adrb2 as a direct target of tRF^Ser-GCT^. In previous studies, the expression of Adrb2 was decreased in obese patients and up-regulated after weight loss (37). In addition, studies have shown that the expression of Adrb2 is low in high-fat feeding chickens, and it is negatively correlated with the expression of Pparγ in chicken preadipocytes, which may have an inhibitory effect on the formation of chicken fat (38). In this study, we first verified the targeting relationship between tRF^Ser-GCT^ and Adrb2 through the dual luciferase reporting system. Further, we found that overexpression of tRF^Ser-GCT^ resulted in a significant decrease in the mRNA level of Adrb2, providing evidence that tRF^Ser-GCT^ may directly regulate the expression of Adrb2. After that, we investigated the effect of the loss of Adrb2 function on the differentiation of 3T3-L1 preadipocytes by q-PCR and Bodipy fluorescence staining. It was confirmed that Adrb2 knockdown could promote the differentiation and lipid accumulation of 3T3-L1 preadipocytes. This demonstrates that tRF^Ser-GCT^ promotes adipogenesis at least in part by inhibiting Adrb2. *In vivo*, restoration of tRF^Ser-GCT^ expression in Hsd17b10-deficient mice partially rescued adipose tissue accumulation and adipogenic gene expression, further supporting the functional significance of the Hsd17b10–tRF^Ser-GCT^–Adrb2 axis in regulating adipose tissue development.

In summary, we found that tRF^Ser-GCT^ promoted the differentiation of preadipocytes. Subsequently, we confirmed the relationship between tRF^Ser-GCT^ and Adrb2 and confirmed that tRF^Ser-GCT^ promoted the differentiation of preadipocytes by inhibiting the level of Adrb2. Further, we investigated the effects of three subunits of the tRF^Ser-GCT^ cutting enzyme RNase P on the content of tRF^Ser-GCT^, and determined that the activity of RNase P could regulate the expression level of tRF^Ser-GCT^. The findings of this study reveal a new Hsd17b10/tRF^Ser-GCT^/Adrb2 axis involved in the progression of fat formation. These results provide new insights into the regulatory network of mitochondrial tRFs in adipose tissue biology.

## Experimental procedures

### Animals and treatments

Eight-week-old male C57BL/6 mice were obtained from DOSSY Experimental Animals Co., Ltd . All mice were maintained in the pathogen-free environment with controlled temperature and lighting, following a 12-h light/12-h dark schedule. To induce obesity *via* a high-fat diet, C57BL/6 mice were randomly allocated to receive either a standard diet or a 60% high-fat purified diet (XTHF60) for duration of 12 weeks. After 2 weeks of high-fat adaptive feeding, the mice were injected of 1 × 10^12^ vg AAV9- Hsd17b10 and AAV control or Agomir-tRF^Ser-GCT^ and Agomir-NC (The sequence provided in Table S1). All adipose tissue were rapidly stored in liquid nitrogen after collection for subsequent experiments. All experiment animals were approved by the Animal Protection and Ethics Committee of Sichuan Agricultural University ( No. 20240461).

### tsRNA isolation, library preparation and data analysis

Total RNA from BAT, gWAT and iWAT was extracted with RNAiso Plus (9109, Takara) and quality-checked by Agilent 2100. 1 to 2 μg RNA was pretreated using an RNA Pretreatment Kit (Arraystar) to remove 3′-aminoacyl, 2′,3′-cyclic phosphate, 5′-OH and demethylate m^1^A/m^3^C. Treated RNA was ligated with 3′ and 5′ adapters, reverse-transcribed and PCR-amplified; products of 134 to 160 bp were size-selected from PAGE and purified. Libraries were quantified by Agilent 2100 and sequenced on an Illumina NextSeq 500 (50 bp single-end). Reads were adapter-trimmed, filtered and mapped to a tRNA reference; tsRNA subtypes (*e.g.*, tRF-5, tRF-3, tRF-1, tiRNA) were assigned based on mapping positions. After adapter trimming and mapping to the tRNA reference, the length of each mapped tsRNA read was extracted from the alignment. For each tissue, reads were tallied by integer length (14–40 nt), and counts were normalized to the total number of mapped tRNA reads to obtain percentage (or RPM). Length distributions were visualized as histograms with 1-nt bins (bars), and peak positions (*e.g.*, ∼22 nt and ∼31 nt) were identified from these discrete distributions. Length distributions, subtype composition, parental tRNA origin and heatmaps were generated from normalized mapped reads for comparative analyses. Heatmap, volcano plot and venn diagram were plotted by https://www.bioinformatics.com.cn (last accessed on 1st April 2025), an online platform for data analysis and visualization. Differentially expressed mouse genes were screened for putative tRF^Ser-GCT^ targets using RNAhybrid v2.1 (https://bibiserv.cebitec.uni-bielefeld.de/rnahybrid). Predictions were constrained to canonical 7-mer seed pairing (positions 2–8) with no mismatches in the seed, allowing G:U wobbles only outside the seed. Predicted potential genes were retained if MFE ≤ −25 kcal/mol and *p* ≤ 0.05.

### RNA-seq and pathway enrichment analysis

Total RNA from mi-tRF^Ser-GCT^ and mi-NC 3T3-L1 cells was sequenced. Reads were mapped to mm39, counts were obtained per gene, and differential expression was computed with DESeq2 (R 4.3). Genes with |log2FC| ≥ 1 and *p* < 0.05 were considered DEGs. KEGG enrichment, GO enrichment, and GSEA plots were generated using the online platform bioinformatics.com.cn, which provides data-analysis and visualization modules. The OmicShare online tool (http://www.omicshare.com/tools) was used to generate the GSEA plots.

### Cell culture and treatment

3T3-L1 cells (Cat. No. GNM25) were obtained from China National Experimental Cell Resource Sharing Service Platform (NICR). The cells were cultured in DMEM (Gibco) containing 10% FBS (fetal bovine serum) (Gibco) in a 37 °C cell incubator with 5% CO_2_. On day 0 of differentiation, cells were treated with PA (100 μM) by direct addition to the differentiation medium and maintained for 8 days, while control (NC) cells received vehicle only.

### Induced adipogenic differentiation *in vitro*

Cells were cultured in DMEM containing 10% FBS. After cell fusion (D0), the cells were replaced with induction solutions containing insulin (10 μg/ml), IBMX (0.5 mM), dexamethasone (1 μM), and rosiglitazone before induction. Differentiation of adipocytes. On day 2, a maintenance solution containing only insulin (10 μg/ml) was used. Thereafter, the cell culture medium was changed every 2 days until day 8. Oil red O staining was performed on day 8 to confirm the differentiation and maturation of preadipocytes. To simulate the high-fat state *in vitro*, we added PA to differentiated and mature 3T3-L1 cells for 24 h.

### Transfection of mimic, shRNA and plasmid

The transfection test was performed in cultured preadipocytes using the Lipofectamine 3000 reagent (Invitrogen) according to the manufacturer’s instructions. The tRF^Ser-GCT^ mimic, mimic negative control, sh-NC and sh-Prorp/Hsd17b10/Trmt10c were synthesized by GenePharma (GenePharma). Coding sequences of Prorp, Hsd17b10, and Trmt10c were cloned into a pLVX-puro lentiviral vector using BamHI/XbaI restriction sites (pLVX digested with BamHI/XbaI, New England Biolabs). 50 nmol of mimic was transfected into the cells; mimic negative control was used as negative control for the mimic. For differentiation, transfection tests were performed when preadipocytes grew to 90% confluence, and preadipocytes were induced to differentiate after 24 h of transfection. Subsequently, cells were harvested at the indicated time points postinduction.

### H&E staining and TOM20 immunofluorescence

For H&E staining, adipose tissues were fixed in 4% paraformaldehyde, paraffin-embedded and sectioned at 5 μm. H&E staining was performed by standard protocols; hematoxylin-stained nuclei appear dark blue/purple and eosin-stained cytoplasm appears pink.

For mitochondrial staining, Paraffin-embedded adipose tissue sections (5 μm) were deparaffinized in xylene and rehydrated through a graded ethanol series to distilled water. Antigen retrieval was performed in EDTA buffer (pH 9.0) using a microwave-based protocol. After cooling, sections were washed with PBS and blocked with 3% BSA or 10% normal rabbit serum for 30 min at RT (10% serum was used when the primary antibody was goat-derived; otherwise 3% BSA was used). Primary antibodies (appropriately diluted in PBS) were applied and sections were incubated in a humidified chamber at 4 °C overnight. Following three washes in PBS, sections were incubated with fluorescence-conjugated secondary antibodies at RT for 50 min in the dark. (When two primary antibodies of the same host species were used, the stains were performed sequentially.) Nuclei were counterstained with 4′,6-diamidino-2-phenylindole (DAPI) for 10 min, and sections were washed, mounted with anti-fade mounting medium, and imaged using a Nikon inverted fluorescence microscope (scale bar: 50 μm).

### Oil red O staining and bodipy staining

For Oil Red O staining, 3T3-L1 cells were fixed with 4% paraformaldehyde for 30 min. After fixation, cells were stained with 0.5% Oil Red O solution for 15 min. Excess dye was removed by washing with PBS, and lipid droplets were visualized and photographed under a light microscope.

For bodipy staining, cells were fixed in 4% paraformaldehyde (Beyotime) at RT for 1 h. Following fixation, the cells were rinsed 2 to 3 times with PBS and then stained with Bodipy (Thermo Fisher Scientific) for 30 min. After an additional 2 to 3 washes, the nuclei were counterstained with DAPI (Beyotime) for 20 min at RT. Finally, the cells were examined using a fluorescence microscope, and the images were analyzed with ImageJ software (version 1.52a, https://imagej.net/ij/).

### TG and NEFA analysis

For TG quantification, intracellular TG levels were measured using a commercial Triglyceride Assay Kit (Nanjing Jiancheng Bioengineering Institute). TG concentrations were normalized to total protein content, which was determined using a BCA Protein Assay Kit (Beyotime Biotechnology).

For NEFA analysis, intracellular NEFA levels were assessed using a NEFA Assay Kit (Nanjing Jiancheng Bioengineering Institute). NEFA concentrations were likewise normalized to total protein content using the BCA Protein Assay Kit (Beyotime Biotechnology).

### RNA extraction and real-time fluorescence quantification

Extraction of the total RNA was conducted with RNAiso Plus reagent (TaKaRa) according to the instructions. cDNA was syn-thesized with Prime Script RT-PCR Kit (TaKaRa) using 1 μg of total RNA. The amplification of cDNA using the Prime Script TMRT Master Mix (Vazyme) was performed to determine the mRNA levels. The levels of targeted genes were represented by 2^-ΔΔCt^. See Supplementary file for primer information. GAPDH was used as the internal control to determine the relative changes in the target samples.

### DNA extraction and quantification of mtDNA

Extraction of the total DNA was conducted with DNA extraction kit (Beyotime) according to the instructions. After extraction, quantify the DNA concentration using a NanoDrop spectrophotometer, ensuring the A260/A280 ratio is between 1.8 and 2.0 for purity. Set the qPCR cycling conditions as follows: initial denaturation at 95 °C for 3 min, followed by 40 cycles of 95 °C for 15 s (denaturation) and 60 °C for 1 min (annealing and extension). Perform melting curve analysis at 95 °C for 15 s, 60 °C for 1 min, and 95 °C for 15 s to confirm amplification specificity. For data analysis, calculate the ΔCt for each sample using the formula ΔCt = Ct (mtDNA target gene) – Ct (reference gene) and normalize mtDNA content to nuclear DNA content. Statistical comparisons between groups should be made using *t* test.

### Fluorescence In Situ Hybridization (FISH)

The 3′-end FAM-labeled tRF^Ser-GCT^ was synthesized by GenePharma with the sequence provided in Table S1. To evaluate the distribution of tRF^Ser-GCT^ in 3T3-L1 cells, a FISH kit (Shanghai GenePharma Co., Ltd) was utilized. Initially, cell slides or cryosectioned tissues were permeabilized using 0.1% Triton X-100 at 25 °C for 15 min. A blocking solution was subsequently applied to minimize nonspecific binding. The probe working solution was prepared and incubated with the sample at 37 °C for 12 h. After hybridization, cell nuclei were stained with DAPI and examined under a confocal microscope.

### Western blotting

Total protein in cells was extracted using RIPA lysis buffer (Beyotime), and protein concentration was determined using BCA kit (Beyotime). Then, approximately 20 μg of protein was separated using sodium dodecylsulfate poly-acrylamide gel electrophoresis and then transferred onto a poly-vinylidine difluoride membrane (Thermo Fisher Scientific). Membranes were blocked with TBST solution containing 5% skim milk for 3 h at RT, and then incubated overnight at 4 °C with primary antibodies: Hsd17b10 (ABclonal, A15686, 1:1000), HSL (ABclonal, A15686, 1:1000), ATGL (ABclonal, A5126, 1:1000), FABP4 (Abclonal, A0232, 1:1000), GAPDH (ABclone; 1:5000), PPARγ (ABclonal, A19676, 1:1000), CEBPα (ABclonal, A0904, 1:1000), ADRB2 (Abcam, ab182136, 1:1000), PRORP (Proteintech, 20959-1-AP, 1:1000), Trmt10c (Proteintech, 29087-1-AP, 1:1000), Hsd17b10 (Proteintech, 10648-1-AP, 1:1000). The membranes were then incubated with HRP-conjugated secondary antibodies for 1 h at RT. Finally, a high sensitivity ECL luminescence kit (SB-WB004, ShareBio) was used for color development, and a Tanon 5200 chemiluminescent imaging analysis system (Tanon) was used for exposure and image collection.

### Northern blotting

Northern blotting was used to verify the expression level of RNA. Initially, 4 μg of total RNA were heat-denatured at 95 °C for 2 min and then separated on a 12.5% urea acrylamide gel. The RNA was subsequently transferred to a pre-wetted Hybond-N+ nylon membrane (Millipore). After the transfer, the membrane was subjected to UV crosslinking at 120 mJ using a Stratagene UV Stratalinker 1800 (SCIENTZ03-II) and subsequently hybridized with biotin-labeled DNA probes provided by Sangon Biotech. The sequences for these probes are detailed in Table S4.

### RNA immunoprecipitation (RIP) assay

Cells were rinsed with pre-chilled PBS, detached by scraping, and centrifuged at 1000 rpm for 5 min. Subsequently, cells were lysed in 1 ml of lysis buffer supplemented with Recombinant RNase Inhibitor (100 U/ml; Takara) and a protease inhibitor cocktail (PIC; Roche) on ice for 45 min. The lysate was then centrifuged at 12,000*g* for 15 min at 4 °C, and the supernatant was collected for protein quantification using the BCA assay. Samples were incubated with Antibody HSD17B10 (#10648-1-AP, Proteintech) or control IgG (#2729, CST) overnight at 4 °C, then samples were incubated with 30 μg of Dynabeads Protein G magnetic beads (InvitrogenTM) for 1 h at 4 °C. Beadswere washed three times in lysis buffer. RNA was extracted from beads using the Trizol method for qRT-PCR, and a portion was used for IP-WB to detect HSD17B10. The results of retrieved RNAs are presented as a percentage of the input.

### Dual-luciferase reporter system

The recombinant plasmid, tRF^Ser-GCT^ mimic and vector were cotransfected into 3T3-L1 cells using Lipofectamine 3000. Cells were harvested after 48 h and assayed for luciferase activity (11402es, YEASEN). Relative luciferase activity was determined by normalization to luciferase activity.

### Protein–RNA docking

Protein–RNA docking was performed to predict the interaction between tRF-^SerGTC^ and galectin-3. The Hsd17b10 sequence (UniProt ID: O08756) was retrieved from the UniProt database (https://www.uniprot.org). The RNA sequence of tRF^SerGTC^ was obtained from our experimental data. Docking was carried out using the AlphaFold server (https://alphafoldserver.com/), which integrates template-based and free docking methods to predict protein–RNA interactions.

### Statistical analysis

All data was statistically analyzed using GraphPad Prism 8 (https://www.graphpad.com/). *p* values were calculated using Student’s *t* test in two group comparison. Data are presented as the mean ± SD. Differences were deemed statistically significant when *p* < 0.05 (∗*p* < 0.05, ∗∗*p* < 0.01, ∗∗∗*p* < 0.001, ∗∗∗∗*p* < 0.0001 or no significant (ns)).

## Data availability

All data generated or analyzed during this study are included in this article.

## Ethics approval and consent to participate

All experimental animals were ethically approved by the Animal Protection and Ethics Committee of Sichuan Agricultural University (Approval No. 20240461), and all procedures adhered to the welfare and ethical standards set by the university's Animal Management Committee.

The tsRNAs sequencing and RNA sequencing raw data can be obtained in the NCBI database under the accession numbers: SRP439993, SRP619302.

## Consent for publication

All authors have thoroughly reviewed this manuscript and kindly request its exclusive consideration for publication in Journal of Biological Chemistry.

## Supporting information

This article contains supporting information.

## Conflict of interest

The authors declare that they have no conflicts of interest with the contents of this article.

## Figures and Tables

**Supporting Figure S1:**