# Supplementary material for: RNase P generated tRFSer-GCT promotes fat storage in adipocytes via Adrb2 signaling
Source: J Biol Chem. 2025 Oct 14;301(12):110820. doi: 10.1016/j.jbc.2025.110820 (PMC12648613; doi:10.1016/j.jbc.2025.110820)
Supplement: Supporting information [file mmc1.docx]

Supplementary Materials for

**RNase P generated tRF^Ser‑GCT^ promotes fat storage in adipocytes via Adrb2 signaling**

Linyuan Shen *et al.*

*Corresponding author: Li Zhu

**This PDF file includes:**

Supplementary Text

Figs. S1

Tables S1 to S4

**Figure S1. Validation of RNase P subunit manipulation and tRNA-Ser-GCT dependency in tRF^Ser-GCT^ biogenesis.** A-C, qRT-PCR validation of knockdown efficiency for Hsd17b10 (A), Trmt10c (B), and Prorp (C). D-F, qRT-PCR validation of overexpression efficiency for Hsd17b10 (D), Trmt10c (E), and Prorp (F). G-I, qRT-PCR validation of the interdependence among RNase P subunits after knockdown of each subunit. J, M, RT-PCR analysis showing that knockdown or overexpression of tRNA^Ser-GCT^ directly affects tRF^Ser-GCT^ levels. K, N, qPCR, analysis showing that knockdown or overexpression of tRNA^Ser-GCT^ directly affects tRF^Ser-GCT^ levels. L, O, Northern blot analysis showing that knockdown or overexpression of tRNA^Ser-GCT^ directly affects tRF^Ser-GCT^ levels.

**Table S1. The sequences of RNA oligonucleotide and FISH probe**

| **Names** | **Sequence 5’-3’** |
| --- | --- |
| Agomir-NC | F: CUCCGAACGUGUCACGUTT |
|  | R: CGUGACACGUUCGGAGAATT |
| Agomir-tRF^Ser-GCT^ | F: AAGAAAGAUUGCAAGAACUGCUAAUUCAU |
|  | R: GAAUUAGCAGUUCUUGCAAUCUUUCUUUU |
| AAV control | F: ATCCGTTCTCCGAACGTGTCACGTAATTCAAGAGATTACGTGACACGTTCGGAGAATTTTTTC |
|  | R: AATTGAAAAAATTCTCCGAACGTGTCACGTAATCTCTTGAATTACGTGACACGTTCGGAGAACG |
| AAV9- Hsd17b10 | F: AATTCGAACCCATTCTTGAATGGActcgagTCCATTCAAGAATGGGTTCTTTTTTG |
|  | R: GATCCAAAAAAGAACCCATTCTTGAATGGActcgagTCCATTCAAGAATGGGTTCG |
| mi-NC | UUCUCCGAACGUGUCACGUTT |
| mi-^tRFSer-GCT^ | AAGAAAGAUUGCAAGAACUGCUAAUUCAU |
| FISH probe-tRF^Ser-GCT^ | ATGAATTAGCAGTTCTTGCAATCTTTCTT |

**Table S2. The sequences of short hairpin RNA (shRNA)**

| **Names** | **Sequence 5’-3’** |
| --- | --- |
| sh-NC | Forward: CCGGCCTAAGGTTAAGTCGCCCTCGCTCGAGCGAGGGCGACTTAACCTTAGGTTTTTG |
|  | Reverse: AATTCAAAAACCTAAGGTTAAGTCGCCCTCGCTCGAGCGAGGGCGACTTAACCTTAGG |
| sh-Trmt10c-1 | Forward: CCGGCGCCTCAACCAGAGAACTAATCTCGAGATTAGTTCTCTGGTTGAGGCGTTTTTG |
|  | Reverse: AATTCAAAAACGCCTCAACCAGAGAACTAATCTCGAGATTAGTTCTCTGGTTGAGGCG |
| sh-Trmt10c-2 | Forward: CCGGATAGGAAGAGAAGTCATTTATCTCGAGATAAATGACTTCTCTTCCTATTTTTTG |
|  | Reverse: AATTCAAAAAATAGGAAGAGAAGTCATTTATCTCGAGATAAATGACTTCTCTTCCTAT |
| sh-Trmt10c-3 | Forward: CCGGGCACCTTCTTTGTCTTGTCATCTCGAGATGACAAGACAAAGAAGGTGCTTTTTG |
|  | Reverse: AATTCAAAAAGCACCTTCTTTGTCTTGTCATCTCGAGATGACAAGACAAAGAAGGTGC |
| sh-Hsd17b10-1 | Forward: CCGGGCTCATCTGGTACAGACCATACTCGAGTATGGTCTGTACCAGATGAGCTTTTTG |
|  | Reverse: AATTCAAAAAGCTCATCTGGTACAGACCATACTCGAGTATGGTCTGTACCAGATGAGC |
| sh- Hsd17b10-2 | Forward: CCGGGTTAGGAGAAAGCTGCATATTCTCGAGAATATGCAGCTTTCTCCTAACTTTTTG |
|  | Reverse: AATTCAAAAAGTTAGGAGAAAGCTGCATATTCTCGAGAATATGCAGCTTTCTCCTAAC |
| sh- Hsd17b10-3 | Forward: CCGGGCTGTCAACTGTGCAGGTATTCTCGAGAATACCTGCACAGTTGACAGCTTTTTG |
|  | Reverse: AATTCAAAAAGCTGTCAACTGTGCAGGTATTCTCGAGAATACCTGCACAGTTGACAGC |
| sh-Prorp-1 | Forward: CCGGATTCCGCAGAGTGGGATAAACCTCGAGGTTTATCCCACTCTGCGGAATTTTTTG |
|  | Reverse: AATTCAAAAAATTCCGCAGAGTGGGATAAACCTCGAGGTTTATCCCACTCTGCGGAAT |
| sh- Prorp -2 | Forward: CCGGAGGAATTGATAGGTCATAATCCTCGAGGATTATGACCTATCAATTCCTTTTTTG |
|  | Reverse: AATTCAAAAAAGGAATTGATAGGTCATAATCCTCGAGGATTATGACCTATCAATTCCT |
| sh- Prorp -3 | Forward: CCGGGTTTCCTAAGGGTCGTGAATCCTCGAGGATTCACGACCCTTAGGAAACTTTTTG |
|  | Reverse: AATTCAAAAAGTTTCCTAAGGGTCGTGAATCCTCGAGGATTCACGACCCTTAGGAAAC |
| sh-tRNA12-SerGCT-1 | Forward: CCGGATTCATGCTTCCATGTTTAAACTCGAGTTTAAACATGGAAGCATGAATTTTTTG |
|  | Reverse: AATTCAAAAAATTCATGCTTCCATGTTTAAACTCGAGTTTAAACATGGAAGCATGAAT |
| sh-tRNA12-SerGCT-2 | Forward: CCGGAGATTGCAAGAACTGCTAATTCTCGAGAATTAGCAGTTCTTGCAATCTTTTTTG |
|  | Reverse: AATTCAAAAAAGATTGCAAGAACTGCTAATTCTCGAGAATTAGCAGTTCTTGCAATCT |
| sh-tRNA12-SerGCT-3 | Forward: CCGGGATTGCAAGAACTGCTAATTCCTCGAGGAATTAGCAGTTCTTGCAATCTTTTTG |
|  | Reverse: AATTCAAAAAGATTGCAAGAACTGCTAATTCCTCGAGGAATTAGCAGTTCTTGCAATC |
| sh-tRNA12-SerGCT-4 | Forward: CCGGTAATTCATGCTTCCATGTTTACTCGAGTAAACATGGAAGCATGAATTATTTTTG |
|  | Reverse: AATTCAAAAATAATTCATGCTTCCATGTTTACTCGAGTAAACATGGAAGCATGAATTA |

**Table S3. The primer sequences for qRT-PCR assay**

| **Names** | **Primer** | **Sequence 5’-3’** |
| --- | --- | --- |
| Prorp | Forward | GATCACGCTAGCATGGACGA |
|  | Reverse | GGAGGGTCGACAAACGAAGT |
| Hsd17b10 | Forward | ACCCTTCCAGAGAAAGTGCG |
|  | Reverse | GCCCTTTAAGGCTGCATTCG |
| Trmt10c | Forward | GCAGTTTGGACAGCCTTTGG |
|  | Reverse | GTGCGCACATTTAAGGAGCC |
| Fabp4 | Forward | ACACCGAGATTTCCTTCAAACTG |
|  | Reverse | CCATCTAGGGTTATGATGCTCTTCA |
| Hsl | Forward | ACCTGAGGCCTTTGAGATGC |
|  | Reverse | GCCAGGCTGTTGAGTACCTT |
| Fasn | Forward | GCTGCGGAAACTTCAGGAAAT |
|  | Reverse | AGAGACGTGTCACTCCTGGACTT |
| Srebp-1c | Forward | TGACCCGGCTATTCCGTGA |
|  | Reverse | CTGGGCTGAGCAATACAGTTC |
| Pparγ | Forward | CATTCGCATCTTTCAGGG |
|  | Reverse | GGACGCCATACTTTAGGA |
| Scd1 | Forward | CCGGAGACCCCTTAGATCGA |
|  | Reverse | TAGCCTGTAAAAGATTTCTGCAAACC |
| Ces1d | Forward | CCCCTGGTCCACAACAGAAG |
|  | Reverse | ACAGGTGGTGAGGATGGGTA |
| Adrb2 | Forward | CTGGTTGGGCTACGTCAACT |
|  | Reverse | CTTCCTTGGGAGTCAACGCT |
| Slc36a2 | Forward | TGTCGGGCACCAAGCATAAT |
|  | Reverse | GGGTGGAAGCCATCATCGAAT |
| Elovl3 | Forward | AGTACTGGTGAGACCTGGTGA |
|  | Reverse | TTGCTTGAGGCCCACTGTAA |
| Cebpα | Forward | CAAGAACAGCAACGAGTACCG |
|  | Reverse | GTCACTGGTCAACTCCAGCAC |
| β-actin | Forward | GGCTGTATTCCCCTCCATCGA |
|  | Reverse | CCAGTTGGTAACAATGCCATGT |
| tRF^Ser-GCT^ | Forward | AAGAAAGATTGCAAGAACTGCTAATTCAT |
|  | Reverse | Uni-miR qPCR Primer, included in kit (TaKaRa) |
| U6 | Forward | CTCGCTTCGGCAGCACA |
|  | Reverse | AACGCTTCACGAATTTGCGT |

**Table S4. Northern blot probe sequences.**

| Gene name | Sequence (5'-3') |
| --- | --- |
| Biotin-U6 | TGGAACGCTTCACGAATTTG |
| Biotin-tRF^Ser-GCT^ | ATGAATTAGCAGTTCTTGCAATCTTTCTT |
| Biotin-tRNA^Ser-GCT^ | TGGTAAGAAAGCCATGTTTTTAAACATGGAAGCATGAATTAGCAGTTCTTGCAATCTTTCTT |
